# The Influence of the Parameters of a Gold Nanoparticle Deposition Method on Titanium Dioxide Nanotubes, Their Electrochemical Response, and Protein Adsorption

**DOI:** 10.3390/bios9040138

**Published:** 2019-11-20

**Authors:** Ewa Paradowska, Katarzyna Arkusz, Dorota G. Pijanowska

**Affiliations:** 1 Department of Biomedical Engineering, Faculty of Mechanical Engineering, University of Zielona Gora, Prof. Z. Szafrana 4, 54-516 Zielona Gora, Poland; k.arkusz@ibem.uz.zgora.pl; 2 Nalecz Institute of Biocybernetics and Biomedical Engineering, Polish Academy of Sciences, Ks. Trojdena 4, 02-109 Warszawa, Poland; dpijanowska@ibib.waw.pl

**Keywords:** titanium dioxide nanotubes, gold nanoparticles, deposition process, cyclic voltammetry, electrochemical characteristics, protein adsorption

## Abstract

The goal of this research was to find the best conditions to prepare titanium dioxide nanotubes (TNTs) modified with gold nanoparticles (AuNPs). This paper, for the first time, reports on the influence of the parameters of cyclic voltammetry process (CV) -based AuNP deposition, i.e., the number of cycles and the concentration of gold salt solution, on corrosion resistance and the capacitance of TNTs. Another innovation was to fabricate AuNPs with well-formed spherical geometry and uniform distribution on TNTs. The AuNPs/TNTs were characterized using scanning electron microscopy, X-ray photoelectron spectroscopy, electrochemical impedance spectroscopy, and open-circuit potential measurement. From the obtained results, the correlation between the deposition process parameters, the AuNP diameters, and the electrical conductivity of the TNTs was found in a range from 14.3 ± 1.8 to 182.3 ± 51.7 nm. The size and amount of the AuNPs could be controlled by the number of deposition cycles and the concentration of the gold salt solution. The modification of TNTs using AuNPs facilitated electron transfer, increased the corrosion resistance, and caused better adsorption properties for bovine serum albumin.

## 1. Introduction

In recent decades, electrochemical biosensors have been an active research field, attracting considerable attention as potential successors to a wide range of analytical techniques with rapid response and high selectivity [1,2]. Titanium dioxide nanotube arrays have demonstrated a number of important applications, including biosensors for the detection of interleukin-6 [3] or glucose [4]. Titanium dioxide nanotube (TNT) structures can be produced through the anodization of titanium foil [5]. In addition, a recent study by the authors has shown that titanium dioxide nanotube arrays with large surface areas, easy and inexpensive preparation, and chemical and thermal stability arepromising for the immobilization of biomolecules, such as horseradish peroxidase for electrochemical biosensors [2].

The electric conductive and adsorption properties of TNT arrays depend on many factors, e.g., the morphology of the nanotubes (diameter, height) and the modification process. Protein adsorption has been demonstrated for nanotubes with a diameter ranging from 20 nm to 70 nm [6]. The electrical conductivity of TNT arrays can be significantly improved by introducing metal nanoparticles to the surface to facilitate electron transfer [1,7,8,9]. Recently, much attention has been paid to using gold nanoparticles (AuNPs) in biosensor construction. Among the advantages of AuNPs are fast and simple methods of synthesis, low costs of production, the ease of binding with proteins in reaction to a thiol group (-SH), and increased electron transfer between the electrode surface and the analyte [8,9].

Several strategies, such as sputtering, photo reduction, soaking at high temperatures, and electrodeposition methods, have been applied to the deposition of gold nanoparticles on TNTs. Sputtering technology can result in the homogeneous dispersion of AuNPs and leads to the production of a thin layer of nonspherical nanoparticles [10,11]. Photo reduction is a multistage and long-lasting technique [12]. The soaking at high temperature and direct adsorption includes long-lasting processes depending on the size and number of the AuNPs [13,14,15]. These methods depend on many factors that influence the reproducibility of the nanoparticle deposition process. Additionally, AuNP deposition on TNT surfaces through one of the described methods can easily aggregate and have a large diameter. Electrodeposition is one of the easiest techniques for gold nanoparticle synthesis. These methods are characterized by convenient control mechanisms to obtain a homogeneous surface. In this process, Au^3+^ ions from a tetrachloroauric acid (HAuCl_4_) precursor can be reduced to metallic Au and subsequently deposited onto the nanotube surface. The galvanostatic method results in the formation of nanoparticles with a wide-ranging diameter [16,17]. In the case of modification of the TNT arrays with gold nanoparticles during the anodizing process, there is no linear relationship between the increase in concentration of the solution and the amount of gold impregnated on TNTs. It is important to point out that the anodization must be under strictly defined conditions to avoid precipitation of the nanoparticles during TNT array growth [18]. In the chronoamperometric method, gold nanoparticles are not uniformly deposited on the TNT surfaces and can easily form agglomerates [19]. Due to its simplicity and its convenient control mechanisms in obtaining a homogeneous surface, the cyclic volt amperometric deposition method can be applied. However, in the case of the voltammetric method, there is no clear indication of the effect of the number of cycles on the size and number of the deposited nanoparticles. According to Lianghsen et al. [20], an increase in the number of cycles causes only an increase in the number of nanoparticles; on the other hand, according to Babu et al. [21], this also results in an increase in the diameter of the nanoparticles.

Previously developed biosensors based on AuNPs/TNTs have shown improvement in limit of detection (LODs), e.g., bisphenol A [20] and aflatoxin B1 [22]. Improvements in the LOD can be obtained after the formation of AuNPs with a well-formed spherical geometry and uniform distribution onto TNTs. In the literature, neither the influence of formation conditions of AuNPs through cyclic voltammetry (the number of CV cycles and the concentration of the gold salt solution) nor the effect of the concentration and size of gold nanoparticles on the capacitance of TNT layers has been reported. In this paper, AuNPs loaded onto TNT arrays prepared using the cyclic voltammetry method with a different number of cycles of deposition and different concentrations of gold salt solutions are described. The aim of our research was to compare the impact of the deposition process parameters and the number of deposited cycles of gold (8–80) on the AuNPs’ diameter and agglomerate formation as well as the electrical conductivity of the developed platforms. The effects of the loading of AuNPs on surface morphology, electrical properties, and corrosion resistance were explored. To confirm the possibility of using AuNP-modified TNTs as sensing platforms for the label-free evaluation of protein adsorption, a series of experiments was carried out.

## 2. Experimental

### 2.1. Chemicals and Reagents

Titanium foil (purity 99.7%) 0.25 mm thickness, ethylene glycol (assay 99.8%), ammonium fluoride NH_4_F, bovine serum albumin (BSA, purity ≥98%), phosphate buffered saline (0.01 M PBS, 0.0027 M potassium chloride, and 0.137 M sodium chloride, pH 7.4), and gold (III) chloride hydrate HAuCl_4_∙3H_2_O (assay 99.995%) were purchased from Sigma-Aldrich (St. Louis, MO, USA) and used as supplied. All of the chemicals were of analytical grade and used without further purification. Electrochemical experiments were carried out at room temperature.

### 2.2. Preparation and Thermal Modification of Ti/TNT Arrays

Titanium dioxide nanotubes were prepared by electrochemical anodization of titanium foil in ethylene glycol solution and ammonium fluoride additive [3]. Titanium sheets were cut into 5 mm (width) × 15 mm (height) × 0.25 mm (thickness) and then sonicated in acetone, distilled water and dried under nitrogen. Anodization was performed in ethylene glycol electrolyte (85 wt%) containing ammonium fluoride (0.65 wt%) under potentiostatic conditions at 17 V (Autolab PGSTAT302N Metrohm Herisau, Switzerland) for 3750 s at room temperature. This process was carried out using a two-electrode system with a platinum sheet as a counter electrode and titanium foil as a working electrode, with the anodization surface of 5 mm × 5 mm × 0.25 mm. TNT layers were annealed in the AMP furnace (AMP, Zielona Gora, Poland) under argon atmosphere at 450 °C for 2 h with the heating and cooling rate of 6 °C·min^−1^.

### 2.3. The Electrodeposition of Gold Nanoparticles on Titanium Dioxide Nanotube Arrays

Electrodeposition was performed in a standard three-electrode system with TNTs as the working electrode (5 mm × 5 mm × 0.25 mm), the standard silver/silver chloride electrode (*E*_Ag_*_/_*_AgCl_ = 0.222 V) as a reference electrode, and a platinum sheet counter electrode, with the use of cyclic voltammetry scan from −1.25 V to −0.7 V (versus Ag/AgCl) with a scan rate of 0.05 V/s in a 3 mL of 0.01 M PBS (pH 7.4) containing tetrachloroauric acid. The process was carried out for different number of cycles (8, 20, 40, 60, 80) in 0.1 mM solution of HAuCl_4_ and next for 8, 20, and 40 cycles in different concentration of HAuCl_4_—1 mM, 5 mM, and 10 mM. After deposition, samples were washed with distilled water and dried under nitrogen atmosphere.

### 2.4. Deposition of Bovine Serum Albumin onto AuNPS/TNTs

Bovine serum albumin was dissolved in 0.01 M PBS (pH 7.4) at a concentration of 1 mg/mL. 5 µL of BSA solution was deposited on the surfaces of the TNTs without and with gold nanoparticles for 30 min at 40 ˚C. The efficiency of BSA immobilization on the TNT arrays and the AuNPs/TNT arrays was analyzed using relative change in the values of electrochemical parameters, expressed in percentage form.

### 2.5. Surface Characterization and Electrochemical Measurements

Scanning electron microscopy (FESEM, JEOL JSM-7600F, Tokyo, Japan) and energy-dispersive X-ray spectroscopy (EDS, INCA, Oxford Instruments, Oxford, UK) were used to investigate surface morphology and chemical composition.

XPS analyses were carried out in a PHI Versa Probe II Scanning XPS system using monochromatic Al Kα (1486.6 eV) X-rays focused to a 100 µm spot and scanned over the area of 400 µm × 400 µm. The photoelectron take-off angle was 45° and the pass energy in the analyzer was set to 46.95 eV. Deconvolution of spectra was carried out using the PHI MultiPak software (v.9.9.0.8). Spectrum background was subtracted using the Shirley method.

Open-circuit potential (OCP) measurements and electrochemical impedance spectroscopy (EIS) scans for Ti/TNTs and AuNPs/TNTs samples were annealed in 450 °C in argon atmosphere, and after BSA adsorption, recorded using a standard three-electrode configuration with titanium dioxide nanotubes before and after modification by gold nanoparticles. OCP measurements were carried out at room temperature (25 ± 2 °C) for 1800 s. EIS spectra were performed over a frequency range from 0.1 to10^5^ Hz with the signal amplitude of 0.01 V. All measurements (OCP, EIS) were recorded in PBS solution (0.01 M, 20 mL, pH 7.4).

All the measurements were repeated three times (for three samples, n = 3). The results of electrochemical studies in the Bode and Nyquist representation show the curves for measuring the closest to the average value of the three samples. In order to select the equivalent circuit the Nova 2.1.4 software was used.

The values of standard deviation (SD) and relative standard deviation (RSD, presented in Appendix A—Table A1, Table A2) for electrochemical parameter measurements (OCP, EIS) were calculated, and equations shown in (1), (2) below were used, where Χi stands for each of the values of the data for three samples, Χ for the mean of Χi, and n for the number of data points. For the calculation of the SD values for gold nanoparticles diameter, the number of analyzed nanoparticle measurements was 500.

(1) $$\mathrm{SD}\;=\;\sqrt{\frac{\sum_{i\;=\;1}^{n}{\left(Χi-Χ\right)}^{2}}{n-1}}$$

(2) $$\mathrm{RSD}\;=\;\frac{SD}{Χ}$$

All tests were carried out with the use of Autolab (Metrohm) PGSTAT 302N potentiostat/galvanostat.

## 3. Results and Discussion

For the purpose of this study, the electrochemical parameters of titanium dioxide nanotubes with a diameter of 50 ± 5 nm and height of 1000 ± 100 nm, annealed at 450 °C in argon atmosphere for 2 h before and after deposition of gold nanoparticles using cyclic voltammetry (potential from −1.25 V to −0.7 V) [20] for different number of cycles (8, 20, 40, 60, 80) in 0.1 mM HAuCl_4_ were examined. Further analysis compared the titanium dioxide nanotube electrochemical properties in terms of selected number of cycles (8, 20, 40) and different concentration of HAuCl_4_ (0.1 mM, 1 mM, 5 mM, 10 mM). The analysis included comparison of the gold nanoparticles with similar diameter but deposited at different number of cycles and different concentrations of tetrachloroauric acid solution. To confirm the possibility of using this platform as a biosensor, BSA protein detection was carried out.

### 3.1. Characterization of TNTs before and after AuNPs Deposition–Influence of the Number of Cycles

**Figure 1:**
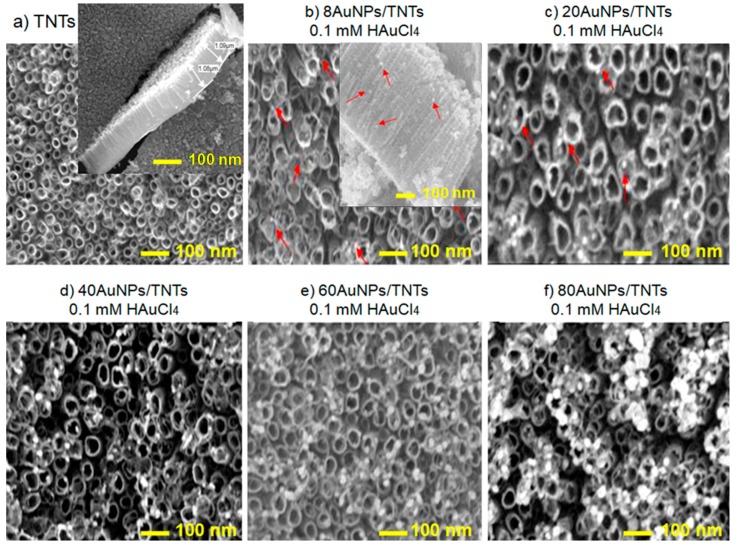
SEM images of TNTs (**a**) after thermal modification anddeposition of AuNPs using CV method carried out in 0.1 mM HAuCl_4_ for (**b**) 8 cycles with cross section, (**c**) 20, (**d**) 40, (**e**) 60, and (**f**) 80 cycles.

Figure 1a shows SEM micrographs of the surface and cross-section of TNT arrays prepared by anodic oxidation at 17 V in NH_4_F/ethylene glycol/H_2_O electrolyte solutions, annealed in argon according toa process described in Section 2.1. The TNT arrays with diameter of 50±5 nm and height of 1000 ± 100 nm had smooth walls without any perforation and were uniformly arranged on the titanium foil. No damage on the TNT layers after annealing at 450°C for 2 h was observed. Thermal modification at 450°C in argon atmosphere for 2 h TNTs enables changing of TiO_2_ from amorphous form (originally present in nanotubes) into crystalline form of rutile and/or anatase [23,24]. The most important advantage of annealing is formation of oxygen vacancies, resulting in the improvement of nanotubes conductivity and thus, facilitating the transfer of electrons attributed to the conversion of Ti^4+^ to Ti^3+^ [25,26]. It was suggested that thermal modification of Ti/TNTs carried out at 450 °C results in the predominance of anatase in their structure [2], which has a higher affinity to biomolecules.

Figure 1b–f shows the SEM images of morphology of Ti/TNTs after deposition of gold nanoparticles. The samples were denoted correspondingly as xAuNPs/TNTs, where x is number of cycles of the deposition process, and x = 8, 20, 40, 60, 80. An [AuCl_4_]^—^ electrolyte solution can be ionized as seen in Equation (3). Au^3+^ near the titanium dioxide nanotube arrays can receive electrons and be reduced to Au, according to the Equation (4) [19]:

[AuCl_4_]^—^ = Au^3+^ + 4Cl^−^ (3)

Au^3+^ + 3e^−^ = Au (4)

Mass transfer in solution occurs by diffusion, migration, and convection, whereas the diffusion and migration result from gradient and electrochemical potential difference respectively, and convection results from an imbalance of forces on the solution. Decrease of Au^3+^ concentrations around the TNTs causes the occurrence of the concentration gradient between the bulk solution and the TNTs. Therefore, Au^3+^ ions move towards the polarized TNTs surface. As a consequence, more reduced Au crystals are formed on the surface of TNT arrays [19].

The AuNPs are homogeneously distributed on the surface of the TNT arrays. Due to higher current densities, nucleation mostly takes place at the boundaries between titanium dioxide nanotubes (Figure 1b–f)—places of the nanotubes contact [19,27]. Countless boundaries (Figure 1a) provided many points of Au nanoparticles nucleation. The homogeneity of TNT arrays provides a homogeneous environment for nucleation of Au nanoparticles [27]. As can be seen in the Figure 1e,f, the possibility of nanoparticles aggregation increases with the increase of the number of cycles.

To enhance the electrochemical responses, the size of nanoparticles should be small and homogeneously dispersed on TNT arrays.

**Figure 2:**
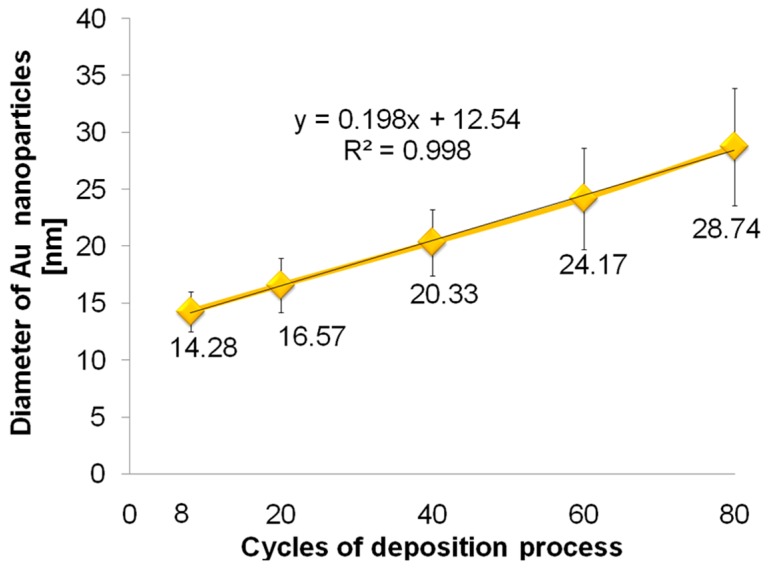
The average values of AuNPs diameter with SD formed and deposited onto TNT susing CV method carried out in 0.1 mM HAuCl_4_ for different number of cycles.

Table 1 shows Au components on the surface through EDS analysis in three different places (mean value with SD). Due to low content of gold nanoparticles on the Ti/TNTs surface for the deposition process carried out for 8 and 20 cycles the resulting value of EDS were affected by high measurement error; therefore, it has not been included in Table 1. The result revealed that the loading amount of Au gradually increases with the increase of the number of deposition cycles from 1.42 ± 0.21 wt% for 40 cycles to 3.59 ± 0.18 wt% for 80 cycles. This result is consistent with the results described by Lianghsen et al. [20]. Additionally, the increase in the number the deposition cycles causes the increase of the diameter of deposited gold nanoparticles from 14.3 ± 1.8 nm for 8 cycles to 28.7 ± 5.2 nm for 80 cycles, similarly to Babu et al. [21]. A linear growth (*R*^2^ = 0.998) between the number of CV cycles and diameter of gold nanoparticles deposited on the TNT arrays (Figure 2) may be observed. However, increase of the number of cycles causes high deviation of gold nanoparticles diameter—a higher value of SD. According to Mahmud et al. [28], AuNPs deposition on annealed TNTs surface compared to non-annealed TNTs surface promotes agglomeration around the pore of the titanium dioxide nanotubes with a rather poor size distribution.

Table 1 shows the open-circuit potential average values for annealed TNTs samples before and after Au nanoparticles deposition process. After the AuNPs deposition, OCP of the samples is further enhanced compared to non-modified TNT arrays**.** Au nanoparticles deposited during eight cycles of cyclic voltammetry, indicating the lowest content of gold (based on EDS analysis), caused no change in the OCP value compared to TNTs. AuNPs deposition carried out for 20–80 cycles causes the general trend for the OCP values to increase. It can be explained by two factors, i.e., the homogeneous AuNPs distribution on the surface of the TNT arrays and the inherent inertness of gold [19]. For AuNPs/TNTs, deposition in cyclic voltammetry process carried out for 20–80 cycles, a positive charge of the surface was observed. The negatively charged protein molecules are easily attracted to the positively-charged matrix, which might be used in construction of the biosensing platforms.

**Figure 3:**
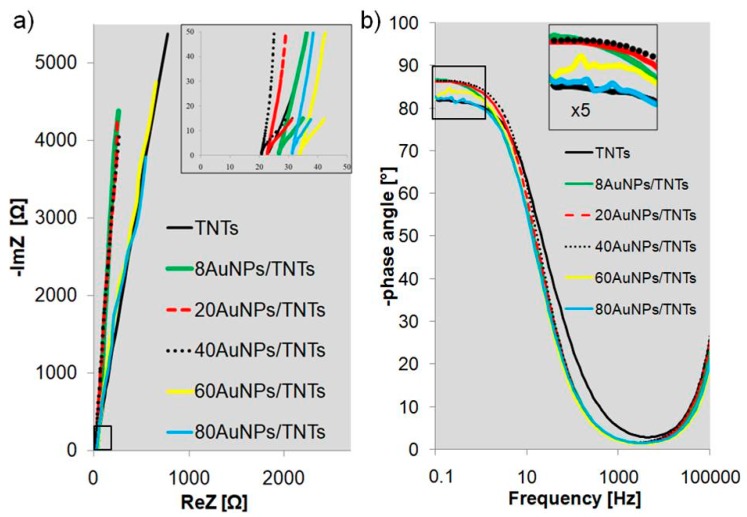
(**a**) Nyquist and (**b**) Bode plots for annealed TNT arrays before and after modification with AuNPs (n = 3) for different number of cycles.

The Nyquist diagrams (Figure 3a) determined for the titanium dioxide nanotube layers before and after the deposition of nanoparticles present fragments of wide, incomplete semicircles characteristic of thin oxide layers [29]. The values recorded in the lowest frequency (0.1 Hz) presented in Table A1 and Figure 3, show that the electrochemical parameters depend on the diameter of Au nanoparticles. Due to good electrical conductivity of the AuNPs, the impedance modulus of TNTs decreases. For 60 and 80 cycles, the impedance modulus value slightly increases. This may result from the formation of agglomerates on the TNTs surface (nanoparticles with a diameter in the range from 24.2 ± 4.4 nm to 28.7 ± 5.2 nm). As presented in Figure 1e,f, many of the nanoparticles are deposited on the inner surface of the titanium dioxide nanotubes, which causes their partial blockage. In addition, the ratio of the gold nanoparticle surface area to their volume is reduced, thus their electrochemical responses deteriorate. It can be noticed that the real impedance (ReZ) value of the xAuNPs/TNTs decreases when compared to non-modified TNT layers. However, this value increases with the increase of the gold nanoparticle diameters. The lowest impedance value 4066 ± 94 Ω and imaginary impedance 4051 ± 97 Ω was noted for nanoparticles with a diameter of 20.3 ± 2.9 nm (40AuNPs/TNTs). These samples have the lowest value of SD and RSD for each of the determined electrochemical parameters. The phase angle values presented in Bode plots (Figure 3b, Table A1) recorded in the lowest frequency (0.1 Hz) are related to the heterogeneity of the sample surface. The lowest heterogeneity value of the phase angle (86.7 ± 0.2°) was observed for the 8AuNPs/TNT layers with the smallest gold nanoparticles (Ø: 14.3 ± 1.8 nm). Heterogeneity of the analyzed structures increases with the increase of the gold nanoparticle diameters from 14.3 ± 1.8 nm for 8AuNPs/TNTs, to 28.7 ± 5.2 nm for 80AuNPs/TNTs.

The equivalent circuit allows for good agreement between experimental data and simulated impedance plots for comparative estimation of specific components of the studied surfaces.

**Figure 4:**
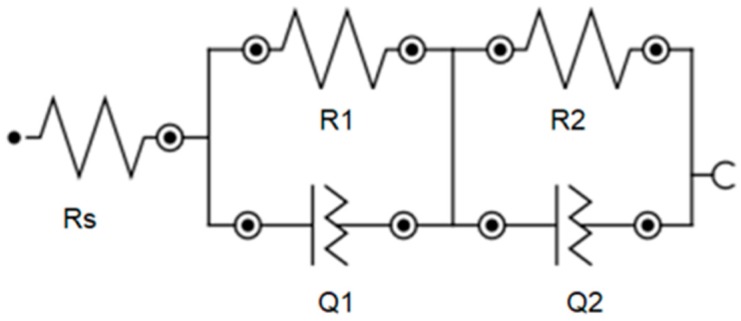
Equivalent circuit used to model the impedance spectra of the TNTs and AuNPs/TNTs.

The equivalent circuit which corresponds to both the TNT arrays/electrolyte interface and AuNPs/TNTs/electrolyte interface are shown in Figure 4, where Rs stands for resistance between sample and solution, parallel combination R1Q1 represents resistance and constant phase element with the capacitance C1 of the porous TiO_2_, combination R2Q2 determining titanium dioxide nanotubes layer, bare and modified with AuNPs. Due to the TNTs and AuNPs/TNTs surface heterogeneities, a constant phase element Q is used to build a model [6,30,31].

The capacitance values (C1, C2) were calculated according to the equation:

(5) $$C\;=\;\frac{RQ^{1/2}}{R}[F]$$

The electrical parameters obtained by fitting equivalent circuits to the measured data are shown in Table 2. For all electrodes modified by an AuNPs deposition, there is a decrease in charge transfer resistance values when compared with bare TNT, confirming easier electron transfer and presence of deposited gold. The time constant (T) calculated for R2C2 increase with the increased number of deposition cycles, and reaches the maximum value for 60AuNPs/TNTs. The T increase is accompanied by increasing number of gold nanoparticles providing pathways for the electrons. The significant change of time constant recorder for 60AuNPs/TNTs and its further decreasing for 80AuNPs/TNTs indicates more unstable deposition process, i.e., agglomerates formation. Moreover, deposition of AuNPs on TNTs results in the decrease of the Rs, while the biggest change was observed for TNT/AuNPs free of agglomerates, i.e., 8AuNPs/TNTs, 20AuNPs/TNTs, 40AuNPs/TNTs.

As shown in Table A1, Table 1 and Table 2 the lowest value of |Z| was observed for gold nanoparticles deposition in 40 cycles, the lowest RSD for 8, 20, and 40 cycles, OCP close to 0 for 20, and 40 cycles, and minimal values of T providing the best electron transfer for 8, 20, and 40 cycles. That is why for further analysis, including impact assessment of the concentration of tetrachloroauric acid solution on the capacitance and adsorption properties of TNT arrays, the samples 8AuNPs/TNTs, 20AuNPs/TNTs, 40AuNPs/TNTs were selected.

### 3.2. Characterization of TNTs after AuNPs Deposition—Influence of Various Concentrations of Gold Salt Solutions

**Figure 5:**
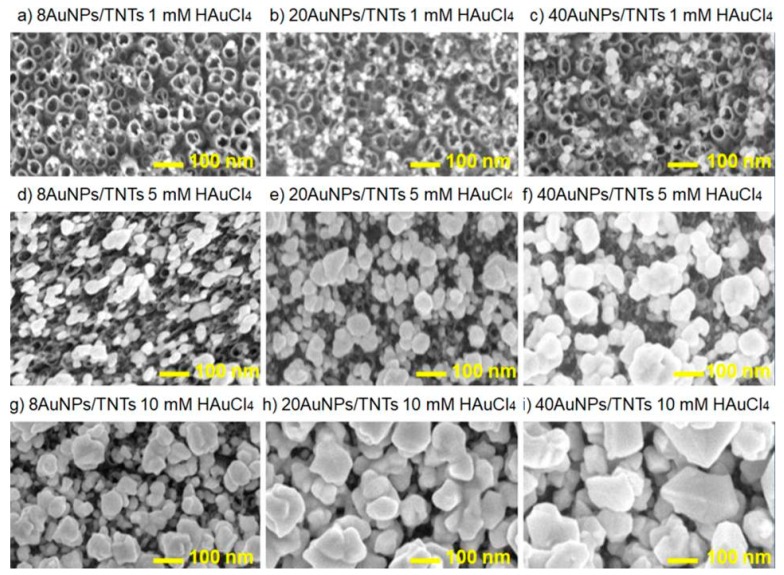
SEM images of annealed TNT arrays after deposition of AuNPs using CV method carried out for different number of cycles 8, 20, 40 (in columns) and in different concentration of HAuCl_4_ solution: (**a**–**c**) 1 mM, (**d**–**f**) 5 mM, (**g**–**i**) 10 mM (in line).

Figure 5a–i shows the results of the microscopic analysis of TNTs surface after the AuNPs deposition process carried out at potential ranging from −1.25 V to −0.7 V and for the selected (in the Section 3.1) number of cycles (8, 20, 40). The cyclic volt amperometry process was carried out in different concentrations of HAuCl_4_ solution (1 mM, 5 mM, 10 mM). The samples were denoted correspondingly as xAuNPs/TNTs, where x = 8, 20, 40 is the number of deposition process cycles.

As can be seen in the microphotographs, the Au nanoparticles were spherical and highly dispersed both outside and inside the surface of TNTs, especially on the top of the nanotubes. Due to high current densities, nucleation mostly takes place at the boundaries between the titanium dioxide nanotubes, which is consistent with the results obtained by Bai et al. [19] and Yang et al. [27]. The amount of AuNPs loaded on TNT arrays surface increased with the increase of the gold salt solution concentration. However, gold nanoparticles deposited in higher concentration of HAuCl_4_ (5 mM, 10 mM) led to production of the nanoparticles with the diameter exceeding the diameter of titanium dioxide nanotubes and formation of many agglomerates (Figure 5d–i). According to Mahmud et al. [28], AuNPs deposition on the annealed TNT arrays compared to non-annealed TNT arrays promotes formation of agglomerates around the pore of the titanium dioxide nanotubes. This is due to the removal of residual ions by thermal modification process, which is unfavorable for dispersion of AuNPs and causes its agglomeration [28]. Additionally, the advantage of using solutions with lower concentration (0.1 mM) is that there is no need of using stabilizers such as polyvinylpyrrolidone [32], which prevents formation of agglomerates. Thus, in order to obtain well-spherical geometry AuNPs with small diameters, it is necessary to use solutions with lower concentration of tetrachloroauric acid.

**Figure 6:**
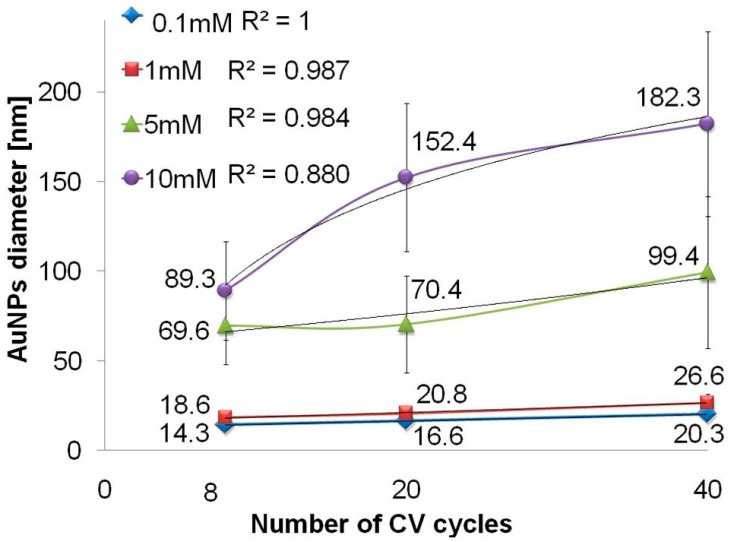
The average values of AuNPs diameters deposited in HAuCl_4_ solution of concentration of 0.1 mM, 1 mM, 5 mM, and 10 mM in different number of cycles (8, 20, and 40). Bars represented a standard deviation, *R*^2^—correlation coefficient.

According to Figure 6, linear growth may be observed between the number of CV cycles and the diameter of gold nanoparticles deposited on the TNT arrays in the lower concentration of gold salt solutions: 0.1 mM (*R*^2^ = 1), 1 mM (*R*^2^ = 0.987). For higher concentrations of HAuCl_4_, i.e., 5 mM and 10 mM, the diameter of AuNPs decreases logarithmically indicating less stable deposition process. Table 3 shows the Au components of the surface through EDS analysis in three different places (average value with standard deviation). The results show that the loading amount of Au gradually increases as the concentration of gold salt solution increases, which was confirmed by Babu et al. [21]. According to Bai et al. [19], TNT arrays modified with gold nanoparticles provide biocompatible environment favorable for cell attachment, which have a typically elongated morphology with an equal amount of gold of about 4 wt%. Deposition of gold nanoparticles in tetrachloroauric acid solution with various concentrations causes the increase of the nanoparticles diameter (Table 3). Thus, the obtained results confirmed that it is possible to control and modify the nanoparticle diameters and the deposited gold amount by varying the concentration of electrolyte and deposition cycles. As the concentration of gold salt solution increases, the possibility of nanoparticles aggregation and its heterogeneity (higher value of SD) increases as well. This reduces the ratio of surface area to the volume of the obtained gold nanoparticles.

Table 3 shows the average OCP values for the samples after gold nanoparticles deposition process using cyclic volt amperometry. Just as increasing the number of cycles, the increase of the tetrachloroauric acid concentration also increases the value of open circuit potential to higher positive values. The obtained results are similar to the ones described in the literature. Bai et al. [19] analyzed TNT arrays after the AuNPs deposition with the use of chronoamperometry method for various time of process. The increase of the amount of the deposited gold causes the increase of the titanium dioxide nanotubes corrosion resistance. High value of open circuit potential was observed for the samples modified with the gold nanoparticles deposited in the solutions of higher concentration (10 mM for 20AuNPs/TNTs and 40AuNPs/TNTs). Electrostatic attraction of oppositely charged protein residues and the electrode surface facilitates the immobilization of the protein in an electro active orientation, further facilitating direct electron transfer between a redox center and the electrode. The application of a potential difference on the electrode can affect the behavior of the proteins on the surface and even its denaturation. For that reason, the electrode potential should be close to zero.

The electrical parameters obtained by fitting equivalent circuits are shown in Table 4. The T calculated for the lower concentration of HAuCl_4_ (0.1 mM, 1 mM) is characterized by minimum value, providing good electron transfer and repeatability of deposition process. Increasing the concentration of HAuCl_4_ results in agglomeration formation and less stability of deposition process confirming the higher value of T. For all electrodes modified by AuNPs deposition, the Rs increased with the increase of concentration of the gold salt solution. Among the analyzed samples, the 40AuNPs/TNTs deposition in 0.1 mM of gold salt solution is characterized by the lowest Rs, confirming the best electrical conductivity of this sample.

Performing a cyclic voltammetry process, in which 0.1 mM solution of tetrachloroauric acid was used as the precursor, leads to the production of small diameter gold nanoparticles and prevents the formation of agglomerates. The homogeneity of the AuNPs/TNTs results in higher repeatability expressed by the lowest values of the relative standard deviations for deposition carried out in 0.1 mM HAuCl_4_ (Table A1 and Table A2). From these samples, 40AuNPs/TNTs is characterized by the lowest Rs and one of the easiest charge transfers (Table 4). This sample is characterized by positive stationary potential (25 ± 8.6 mV) with the value close to 0 V (Table 3), which does not deactivate and promotes protein adsorption [29]. Therefore, for further analysis including XPS and the deposition of biological elements i.e., bovine serum albumin, TNT arrays were chosen deposition of gold nanoparticles by cyclic voltammetry method carried out for 40 cycles in 0.1 mM HAuCl_4_.

**Figure 7:**
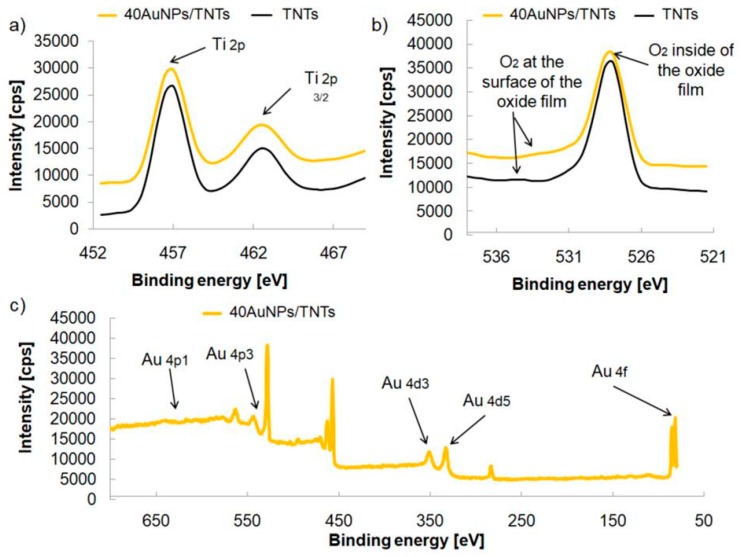
Chemical composition of the annealed TNTs (diameter 50 ± 5 nm, 1000 ± 100 nm thickness) before and after gold nanoparticles deposition measured with XPS: (**a**) Ti 2*p* spectra, (**b**) O 1*s* spectra (**c**) Au spectra.

The results of the XPS analysis of the TNTs before and after modification with gold nanoparticles (40 cycles, 0.1 mM HAuCl_4_) are shown in Figure 7. TiO_2_ and Ti_2_O_3_ were found on the surface of the nanotube layers. The standard binding energy of Ti 2*p*3/2 in TiO_2_ for Ti^3+^ is usually located at 457.7 eV and for Ti^4+^ is at 459.5 eV [33,34]. The O 1*s* binding energy for TiO_2_ is 529.3 eV [34]. The analysis of the XPS depth profile of TNTs and 40AuNPs/TNTs indicates higher amount of oxygen absorbed inside of the oxide film rather than on its surface. Thermal modification of TNTs results in the occurrence of the lack of oxygen on the surface, which proves its deficiency, the presence of oxygen vacancies and results in improved TNTs electrical conductivity [25]. For 40AuNPs/TNTs, the main 4*f*7/2 line is shifted to lower binding energy (83.2 eV), which was found to occur in case of nanoparticles of gold with well spherical geometry [35]. This shift is caused by the initial state effects where spherical NPs have larger fraction of uncoordinated surface atoms reducing their binding energies relative to nanoparticles with large diameter [35].

### 3.3. Evaluation of Experimental Conditions for BSA Protein Adsorption on the TNT and 40AuNPs Arrays

**Figure 8:**
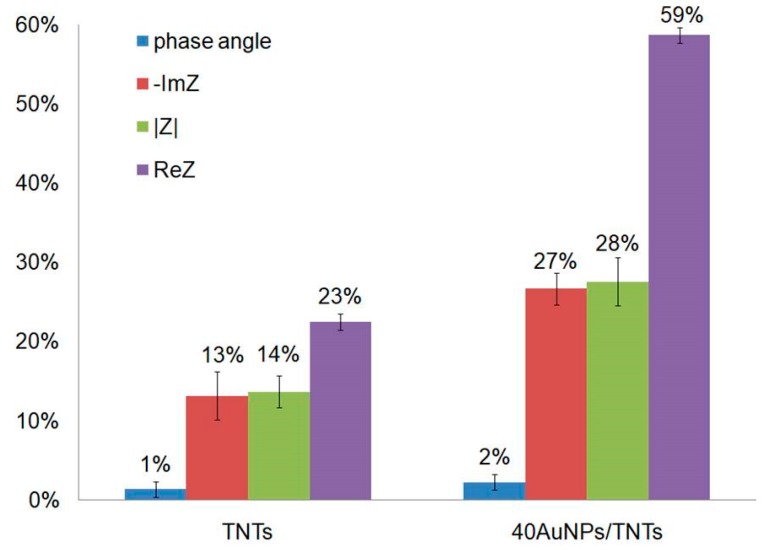
The values of electrochemical parameters with the calculated percentage change in value due to 30 min BSA deposition on TNTs and 40AuNPs/TNT arrays.

Figure 8 shows the results of the electrochemical analysis in Nyquist representation for thermal modification of the TNTs and the AuNPs/TNTs before and after serum bovine albumin immobilization. The immobilization procedure was executed in accordance with Kopac et al. [36], in which BSA deposition was carried out on a double-walled, carbon nanotube (DWCNT) system. For this study, the optimum experimental conditions were observed to be at 40 °C due to the highest value of adsorption efficiency (72%) compared to other temperatures: 25 °C (27%), 30 °C (32%), 37 °C (53%). This showed that the adsorbate onto DWCNT increased with increasing temperature, which, according to [36], can be attributed to the availability of more adsorption sites and increase in the sorptive surface area.

The values of electrochemical parameters presented in Figure 8 obtained for the 40AuNPs/TNTs and the TNT layers before and after the deposition of BSA show that the highest increase in impedance modulus recorded in the lowest frequency (0.1 Hz) by 1247 Ω (28%) was observed for samples modified with gold nanoparticles, while for non-modified titanium dioxide nanotubes, the increase was only 843 Ω (14%).

The electrical parameters obtained by fitting equivalent circuits are shown in Table 5. The equivalent circuit is shown in Figure 4. Deposition of BSA on TNTs and 40AuNPs/TNTs arrays causes an increase in the Rs value. The highest increase in Rs value of 16.29 Ω was recorded for the samples after modification with gold nanoparticles, which confirms the increased adsorption of bovine serum albumin for this platform. For 40AuNPs/TNTs arrays after the BSA deposition process, they are characterized by almost double increase of the T value, which is caused by the increase of electron transfer resistance due to the formation of a protein layer.

Better adsorption of biological elements on the 40AuNPs/TNT arrays results from an increase in the direction of positive OCP values caused by modification of gold nanoparticles. It is very important for the adsorption of BSA, which in the PBS solution (pH 7.4) has negative charge (isoelectric point of BSA protein −5.4). According to several articles on protein immobilization, an appropriate pH for the termination of proteins would be around 7 to 8 [37,38]. During the formation of biological layer on the surface, each adsorbing molecule must go through the following steps:  transport toward the surface, attachment, and another spreading on the surface. According to the electrostatic binding hypothesis, the attraction between the negative surface residues due to the isoelectric point of BSA and the positive charge from the surface are responsible for the strong binding of BSA to gold nanoparticles. In this hypothesis, the protein attaches itself to the passivation layer on the gold surface, with little direct interaction between BSA and the gold surface [38]. According to Liu [39] modification of electrode surfaces with gold nanoparticles provides a microenvironment similar to that of proteins in native systems, and gives the protein molecules orientation freedom. Peng et al. confirmed that the highest titanium dioxide nanotubes promote adsorption and stability of BSA protein binding [40].

## 4. Conclusions

The aim of the described study was to show compare electrochemical properties of TNT arrays before and after modification using gold nanoparticles. AuNPs/TNT platforms were produced using cyclic voltammetry method applying different number of cycles and concentration of gold salt solutions. Due to higher current densities, the nucleation primarily took place at the boundaries between the titanium dioxide nanotubes [19]. Increasing the number of cycles of the deposition process and the concentration of tetrachloroauric acid caused an increase in the diameter of the deposited gold nanoparticles, the amount of the deposited gold, and possibility of nanoparticles aggregation.

The research showed that using spherical and high dispersion on the TNT arrays Au nanoparticles improved the capacitance of the developed platform. Another advantage of AuNPs nanoparticles is improvement of the corrosion resistance of TNTs [19]. The authors determined that the greatest improvement in electrochemical parameters is obtained for nanoparticles that are deposited for 40 CV cycles in 0.1 mM concentration of gold salt solution. Bovine serum albumin adsorption studies confirmed that modification of the TNT arrays with gold nanoparticles promotes the adsorption of biological elements. For samples modified with gold nanoparticles almost double increase of the T value was observed confirming that the AuNPs improve the TNTs adsorption properties. Modification of the TNT surfaces with gold nanoparticles creates a microenvironment similar to that of proteins in native systems and gives the protein molecules freedom in orientation [38]. This study also suggests the possibility of exploring the use of gold nanoparticles to further improve sensitivity of the titanium dioxide nanotubes in label free detection system.

## Figures and Tables

**Table 1:** The average values of OCP, diameter of AuNPs and EDS results (n = 3) with SD measured for thermally modified TNT arrays before and after deposition of AuNPs using CV method carried out in 0.1 mM HAuCl_4_ for different number of cycles (8, 20, 40, 60, 80).

	TNTs	8AuNPs/TNTs	20AuNPs/TNTs	40AuNPs/TNTs	60AuNPs/TNTs	80AuNPs/TNTs
**Au [wt%]**	-	-	-	1.43 ± 0.21	2.35 ± 0.31	3.59 ± 0.18
**AuNPs diameter [nm]**	-	14.3 ± 1.8	16.6 ± 2.4	20.3 ± 2.9	24.2 ± 4.4	28.7 ± 5.2
**OCP [mV] versus Ag/AgCl**	−47 ± 7.2	−48 ± 4.2	20 ± 13.5	25 ± 8.6	40 ± 8.5	49 ± 0.8

**Table 2:** Value of circuit equivalent elements for TNTs and AuNPs/TNT arrays.

Electrical Parameters	TNTs	8AuNPs/TNTs	20AuNPs/TNTs	40AuNPs/TNTs	60AuNPs/TNTs	80AuNPs/TNTs
Rs [Ω]	26.4	10.5	9.91	8.59	11.28	10.74
R1 × 10^3^ [Ω]	5.10	4.90	4.73	4.55	4.95	4.78
C1 × 10^−3^ [F]	1.67	2.16	2.39	2.66	3.46	2.77
N1	0.98	0.99	0.98	0.98	0.98	0.98
R2 × 10^3^ [Ω]	46.10	38.40	35.01	33.70	40.24	37.80
C2 × 10^−4^ [F]	3.59	4.63	4.45	4.72	5.82	4.23
N2	0.94	0.95	0.97	0.98	0.95	0.93
T = R2C2 [s]	16.55	17.78	15.58	15.91	23.42	15.99
Χ^2^	0.0089	0.0079	0.0086	0.0069	0.0055	0.0066

**Table 3:** The average values of EDS analysis, diameter of gold nanoparticles and OCP (n = 3) with SD measured for annealed TNT arrays after deposition of AuNPs using CV method carried out in 0.1 mM, 1 mM, 5 mM, and 10 mM HAuCl_4_ for different number of cycles (8, 20, 40).

	Concentration of HAuCl_4_	8AuNPs/TNTs	20AuNPs/TNTs	40AuNPs/TNTs
Au [wt%]	0.1 mM	-	-	1.43 ± 0.21
	1 mM	1.69 ± 0.16	3.17 ± 0.28	4.06 ± 1.00
	5 mM	16.57 ± 6.16	36.37 ± 1.44	42.58 ± 1.91
	10 mM	49.93 ± 4.61	78.75 ± 1.18	91.98 ± 2.87
AuNPs diameter [nm]	0.1 mM	14.3 ± 1.8	16.6 ± 2.4	20.3 ± 2.9
	1 mM	18.6 ± 3.5	20.8 ± 3.4	26.6 ± 4.8
	5 mM	69.9 ± 21.5 ^a^	70.4 ± 27.0 ^a^	99.4 ± 42.4 ^a^
	10 mM	89.3 ± 27.4 ^a^	152.4 ± 41.4 ^a^	182.3 ± 51.7 ^a^
OCP [mV] versus Ag/AgCl	0.1 mM	−48 ± 4.2	20 ± 13.5	25 ± 8.6
	1 mM	25 ± 7.5	28 ± 3.3	51 ± 2.6
	5 mM	28 ± 8.1	42 ± 15.2	93 ± 1.6
	10 mM	55 ± 2.6	90 ± 0.8	110 ± 10.8

^a^—platform with agglomerates.

**Table 4:** Values of circuit equivalent elements for TNTs before and after gold nanoparticles deposition carried out in different concentration of gold salt solutions (0.1 mM, 1 mM, 5 mM, and 10 mM) and for 8, 20, and 40 cycles.

	Rs [Ω]	R1 × 10^3^ [Ω]	C1 × 10^−3^ [F]	N1	R2 × 10^3^ [Ω]	C2 × 10^−4^ [F]	N2	τ = R2C2 [s]	Χ^2^
**8 cycles**									
**0.1 mM**	10.50	4.90	2.16	0.99	38.40	4.46	0.95	17.78	0.0079
**1 mM**	11.41	5.93	1.69	0.98	43.41	3.99	0.95	17.32	0.0071
**5 mM**	12.52	6.02	1.19	0.98	48.42	2.83	0.96	13.70	0.0064
**10 mM**	21.43	18.91	0.274	0.96	54.46	3.71	0.96	20.20	0.0079
**20 cycles**									
**0.1 mM**	9.91	4.73	2.39	0.98	35.01	4.45	0.97	15.57	0.0086
**1 mM**	14.5	5.63	2.20	0.96	36.01	4.60	0.95	16.56	0.0051
**5 mM**	21.35	20.83	3.75	0.82	63.11	5.56	0.94	35.09	0.0068
**10 mM**	22.89	20.91	6.21	0.78	54.31	5.84	0.94	31.72	0.0081
**40 cycles**									
**0.1 mM**	8.59	4.55	2.66	0.98	33.70	4.72	0.98	15.91	0.0069
**1 mM**	13.45	5.12	2.08	0.98	39.91	4.14	0.98	16.52	0.0065
**5 mM**	21.98	20.13	0.41	0.96	48.41	2.33	0.96	11.28	0.0051
**10 mM**	23.6	20.81	0.78	0.76	24.83	9.24	0.93	22.94	0.0083

**Table 5:** Values of circuit equivalent elements for TNTs and 40AuNPs/TNTs before and after BSA deposition.

		Rs [Ω]	R1 × 10^3^ [Ω]	C1 × 10^−3^ [F]	N1	R2 × 10^3^ [Ω]	C2 [F]	N2	τ = R2C2 [s]	Χ^2^ × 10^−4^
**TNTs**	**Before BSA deposition**	26.42	5.10	1.67	0.98	46.10	0.94	16.55	0.0089	3.59
	**After BSA deposition**	28.91	7.98	0.36	0.95	89.9	0.93	12.68	0.0053	1.41
**40AuNPs/TNTs**	**Before BSA deposition**	8.59	4.55	2.66	0.98	33.70	0.98	15.91	0.0069	4.72
	**After BSA deposition**	24.85	6.95	0.51	0.89	78.72	0.89	32.90	0.0048	4.18

## Appendix A

**Table A1:** Table of mean values of phase angle, impedance modulus (|Z|), real impedance (ReZ) and imaginary impedance (-ImZ) recorded at the lowest frequency of 0.1 Hz, with SD and RSD measured for annealed TNTs before and after the deposition of AuNPs using cyclic voltammetry carried out in 0.1 mM HAuCl_4_ for different number of cycles (8, 20, 40, 60, 80).

	|Z| [Ω]	RSD	ReZ [Ω]	RSD	-ImZ [Ω]	RSD	-Phase Angle [˚]	RSD
**TNTs**	5608 ± 608	0.09	758 ± 58	0.06	5556 ± 556	0.09	82.2 ± 2.2	0.005
**8AuNPs/TNTs**	4576 ± 576	0.05	290 ± 90	0.17	4576 ± 576	0.09	86.7 ± 6.7	0.002
**20AuNPs/TNTs**	4365 ± 365	0.04	**249 ± 49**	0.13	4331 ± 331	0.04	86.2 ± 6.2	0.008
**40AuNPs/TNTs**	**4066 ± 06**	0.02	300 ± 00	0.12	**4051 ± 05**	0.02	85.7 ± 5.7	0.006
**60AuNPs/TNTs**	4609 ± 609	0.09	627 ± 27	0.18	4591 ± 591	0.09	82.0 ± 2.0	0.007
**80AuNPs/TNTs**	4208 ± 208	0.06	556 ± 56	0.22	4315 ± 315	0.06	81.4 ± 1.4	0.006

—the lowest value of RSD.

**Table A2:** Table of mean values of phase angle and |Z| recorded in the lowest frequency (0.1 Hz), resistance ReZ and –ImZ with SD and RSD measured for annealed TNT arrays after deposition of AuNPs using CV method for different number of cycles (8, 20, 40) and concentration of HAuCl_4_ 0.1 mM, 1 mM, 5 mM, 10 mM.

	|Z| [Ω]	RSD	ReZ[Ω]	RSD	-ImZ[Ω]	RSD	-Phase Angle [˚]	RSD
**8 cycles**								
**0.1 mM**	4576 ± 210	0.05	290 ± 50	0.17	4576 ± 394	0.09	86.7 ± 0.2	0.002
**1 mM**	4874 ± 135	0.03	431 ± 161	0.37	4937 ± 125	0.03	85.2 ± 1.6	0.019
**5 mM**	4834 ± 599	0.12	1179 ± 137	0.12	4683 ± 591	0.13	75.8 ± 1.1	0.015
**10 mM**	4776 ± 81	0.02	1282 ± 231	0.18	4596 ± 138	0.03	74.4 ± 3.1	0.040
**20 cycles**								
**0.1 mM**	4365 ± 158	0.04	249 ± 32	0.13	4331 ± 163	0.04	86.2 ± 0.7	0.008
**1 mM**	4549 ± 170	0.04	741 ± 122	0.16	4488 ± 161	0.04	80.6 ± 1.4	0.017
**5 mM**	4830 ± 430	0.09	1373 ± 209	0.15	4632 ± 428	0.09	74.4 ± 2.2	0.030
**10 mM**	2995 ± 444	0.15	1695 ± 74	0.04	2496 ± 420	0.17	55.3 ± 1.9	0.034
**40 cycles**								
**0.1 mM**	4066 ± 94	0.02	300 ± 36	0.12	4051 ± 97	0.02	85.7 ± 0.5	0.006
**1 mM**	4260 ± 180	0.04	485 ± 47	0.10	4510 ± 288	0.06	83.9 ± 0.9	0.011
**5 mM**	3859 ± 158	0.04	938 ± 40	0.04	3743 ± 173	0.05	75.9 ± 1.2	0.016
**10 mM**	2304 ± 98	0.04	1442 ±105	0.07	1796 ± 46	0.03	51.3 ± 1.5	0.029

—the lowest value of RSD

## References

[1] Kang Q., Yang L., Cai Q. (2008). An electro-catalytic biosensor fabricated with Pt-Au nanoparticle-decorated titania nanotube array. Bioelectrochemistry.

[2] Arkusz K., Paradowska E., Nycz M., Krasicka-Cydzik E. (2018). Influence of thermal modification and morphology of TiO_2_ nanotubes on their electrochemical properties for biosensors applications. J. Nanosci. Nanotechnol..

[3] Arkusz K., Paradowska E., Nycz M., Krasicka-Cydzik E. (2014). Electrochemical detection method for interleukin-6 on titania nanotube platforms. Eng. Biomater..

[4] Gao Z.D., Qu Y., Tongtong L., Shrestha N.K., Song Y.Y. (2014). Development of amperometric glucose biosensor based on prussian blue functionlized TiO_2_ nanotube arrays. Sci. Rep..

[5] Portan D., Papaefthymiou K., Pirvu C. (2011). Manufacturing and characterization of tio_2_ nanotubes on pure titanium surfaces for advanced biomedical applications. UPB. Sci. Bull. Ser. B.

[6] Xiao P., Liub D., Garcia B., Sepehri S., Zhang Y., Cao Y.G. (2008). Electrochemical and photoelectrical properties of titania nanotube arrays annealed in different gases. Sens. Actuators B.

[7] Nycz M., Arkusz K., Pijanowska D.G. (2019). Influence of the silver nanoparticles (AgNPs) formation conditions onto titanium dioxide (TiO_2_) nanotubes based electrodes on their impedimetric response. Nanomat.

[8] Luo P.F., Kuwana T. (1994). Nickel-Titanium alloy electrode as a sensitive and stable LCEC detector for carbohydrates. Anal. Chem..

[9] Yu J., Ju H. (2002). Preparation of porous titania sol−gel matrix for immobilization of horseradish peroxidase by a vapor deposition method. Anal. Chem..

[10] Kafi A.K.M., Guosheng W., Aicheng C.H. (2008). A novel hydrogen peroxide biosensor based on the immobilization of horseradish peroxidase onto Au-modified titanium dioxide nanotube arrays. Biosens. Bioelectron..

[11] Low C.T.J., Ponce-de-León C., Walsh F.C. (2012). A gold-coated titanium oxide nanotube array for the oxidation of borohydride ions. Electrochem. Commun..

[12] Huang Q., Gao T., Niu F., Chen D., Chen Z., Qin L., Sun S., Huang Y., Shu K. (2014). Preparation and enhanced visible-light driver photocatalytic properties of Au-loaded TiO_2_ nanotube arrays. Superlattice Micros..

[13] Macak J.M., Schmidt-Stein F., Schmuki P. (2007). Efficient oxygen reduction on layers of ordered TiO_2_ nanotubes loaded with Au nanoparticles. Electrochem. Commun..

[14] Abdelmoula M., Panaitescu E., Phan M., Yin D., Richter C., Lewis L.H., Menon L. (2009). Controlled attachment of gold nanoparticles on ordered titania nanotube arrays. J. Mater. Chem..

[15] Maltanawa H. (2016). Electrocatalytic activity of Au nanoparticles onto TiO_2_ nanotubular layers in oxygen electroreduction reaction: Size and suport effects. Electrochim. Acta.

[16] Hosseini M.G., Faraji M., Momeni M.M., Ershad S. (2011). An innovative electrochemical approach for voltammetric determination of levodopa using gold nanoparticles doped on titanium dioxide nanotubes. Microchim. Acta.

[17] Hosseini M.G., Mome M. (2010). Gold particles supported on self-organized nanotubular TiO_2_ matrix as highly active catalysts for electrochemical oxidation of glucose. J. Solid State Electrochem..

[18] Feil A., Migowski P., Scheffer R., Pierozan M., Corsetti R., Rodrigues M., Pezzi R., Machado G., Amaral L., Teixeira S. (2010). Growth of TiO_2_ nanotube arrays with simultaneous Au nanoparticles impregnation: Photocatalysts for hydrogen production. J. Braz. Chem. Soc..

[19] Bai Y., Bai Y., Wang C., Gao J., Ma W. (2016). Fabrication and characterization of gold nanoparticle-loadedTiO_2_ nanotube arrays for medical implants. J. Mater. Sci. Mater. Med..

[20] Lianghsen H., Fong C.H., Zhang X., Chan L.L., Lam P.K.S., Chu P.K., Wong K., Yang M. (2016). Au Nanoparticles decorated TiO_2_ nanotube arrays as a recyclable sensor for photoenhanced electrochemical detection of bisphenol A. Environ. Sci. Technol..

[21] Babu T.G.S., Varadarajan D., Murugan G., Ramachandran T., Nair B.G. (2012). Gold nanoparticle–polypyrrole composite modified TiO_2_ nanotube array electrode for the amperometric sensing of ascorbic acid. J. Appl. Electrochem..

[22] Qiong Y., Chuxian H., Rijian M., Chengyong L. (2018). Detection of AFB1 via TiO_2_ nanotubes/Au nanoparticles/enzyme photoelectrochemical biosensor. Coatings.

[23] Mor G.K., Varghese O.K., Paulose M., Mukherjee N., Grimes C.A. (2003). Fabrication of tapered, conical-shaped titania nanotubes. J. Mater. Res..

[24] Albu S.P., Tsuchiya H., Fujimoto S., Schmuki P. (2010). TiO_2_ nanotubes-annealing effects on detailed morphology and structure. Eur. J. Inorg. Chem..

[25] Liu F., Lu L., Xiao P., He H., Qiao L., Zhang Y. (2012). Effect of oxygen vacancies on photocatalytic efficiency of TiO_2_ nanotubes aggregation. B Korean Chem. Soc..

[26] Salari M., Aboutalebi S.H., Chidembo A.T., Nevirkovets I.P., Konstantinov K., Liu H.K. (2012). Enhancement of the electrochemical capacitance of TiO_2_ nanotube arrays through controlled phase transformation of anatase to rutile. Phys. Chem. Chem. Phys..

[27] Yang L., Luo S., Su F., Xiao Y., Chen Y., Cai Q. (2010). Carbon-nanotube-guiding oriented growth of gold shrubs on TiO_2_ nanotube arrays. J. Phys. Chem. C.

[28] Mahmud A., Habiballah A. (2015). Synthesis and morphological characterization of gold nanoparticles (AuNPs) supported on anodized titanium oxide (TiO_2_) nanotubes. Mater. Sci. Forum.

[29] Goodarzi S., Moztarzadeh M. (2016). Titanium dioxide nanotube arrays: A novel approach into periodontal tissue regeneration on the surface of titanium implants. Adv. Mater. Lett..

[30] Yu W.Q., Qiu J., Xu L., Zhang F.Q. (2009). Corrosion behaviors of TiO_2_ nanotube layers on titanium in Hank’s solution. Biomed. Mater..

[31] Al-Swayih A. (2014). Electrochemical characterization of titanium oxide nanotubes fabricated by anodizing in three kinds of electrolytes. Life Sci. J..

[32] Daniel M. (2004). Gold nanoparticles: Assembly, supramolecular chemistry, quantum-size-related properties, and applications toward biology, catalysis, and nanotechnology. Chem. Rev..

[33] Bonanni A., Pumera M., Miyahara Y. (2011). Influence of gold nanoparticle size (2–50 nm) upon its electrochemical behavior: An electrochemical impedance spectroscopic and voltammetric study. Phys. Chem..

[34] Zhang Y.G., Li J.L., Yu Y. (2007). Photoelectrocatalytic activity of highly ordered TiO_2_ nanotube arrays electrode for azo dye degradation. Environ. Sci. Technol..

[35] Radnik J., Mohr C., Claus P. (2003). On the origin of binding energy shifts of core levels of supported gold nanoparticles and dependence of pretreatment and material synthesis. Phys. Chem. Chem. Phys..

[36] Kopac T., Bozgeyik K., Flahaut E. (2018). Adsorption and interactions of the bovine serum albumin-double walled carbon nanotube system. J. Mol. Liq..

[37] Vidal C., Juan A., Munoz A. (2010). Adsorption of bovine serum albumin on CoCrMo surface: Effect of temperature and protein concentration. Colloids Surf. B.

[38] Brewer S.H., Glomm W., Johnson M., Knag M.K. (2005). Probing BSA binding to citrate-coated gold nanoparticles and surfaces. Langmuir.

[39] Liu S.Q., Leech D., Ju H.X. (2003). Application of colloidal gold in protein immobilization, electron transfer, and biosensing. Anal. Lett..

[40] Peng L., Mendelsohn A.D., LaTempa T.J., Yoriya S., Grimes C.A., Desai T.A. (2009). Long-Term Small Molecule and Protein Elution from TiO_2_ Nanotubes. Nano Lett..

